# Extensive halogenated organic compound reservoirs and active microbial dehalogenation in Mariana Trench sediments

**DOI:** 10.1093/ismejo/wraf273

**Published:** 2025-12-10

**Authors:** Rulong Liu, Hui Wei, Zhiao Xu, Yuheng Liu, Jiani He, Zhixuan Wang, Li Wang, Min Luo, Jiasong Fang, Federico Baltar, Yunping Xu, Qirui Liang, Liting Huang

**Affiliations:** College of Oceanography and Ecological Science, Shanghai Ocean University, Shanghai 201306, China; College of Oceanography and Ecological Science, Shanghai Ocean University, Shanghai 201306, China; College of Oceanography and Ecological Science, Shanghai Ocean University, Shanghai 201306, China; College of Oceanography and Ecological Science, Shanghai Ocean University, Shanghai 201306, China; College of Oceanography and Ecological Science, Shanghai Ocean University, Shanghai 201306, China; College of Oceanography and Ecological Science, Shanghai Ocean University, Shanghai 201306, China; College of Oceanography and Ecological Science, Shanghai Ocean University, Shanghai 201306, China; College of Oceanography and Ecological Science, Shanghai Ocean University, Shanghai 201306, China; College of Oceanography and Ecological Science, Shanghai Ocean University, Shanghai 201306, China; Laboratory for Marine Biology and Biotechnology, Qingdao National Laboratory for Marine Science and Technology, Qingdao 266003, China; College of Oceanography and Ecological Science, Shanghai Ocean University, Shanghai 201306, China; Department of Functional and Evolutionary Ecology, University of Vienna, Vienna A-1010, Austria; College of Oceanography and Ecological Science, Shanghai Ocean University, Shanghai 201306, China; College of Oceanography and Ecological Science, Shanghai Ocean University, Shanghai 201306, China; College of Oceanography and Ecological Science, Shanghai Ocean University, Shanghai 201306, China

**Keywords:** hadal trench, carbon cycling, halogenated organic compounds, microbiome, metabolic pathway, dehalogenation

## Abstract

The hadal trenches, the deepest regions of the ocean, serve as the final sinks for marine particles and “tunnels” for material exchange between the ocean and Earth's interior. Despite their extreme conditions, the trench sediments contain high content of organic carbon and active microbial carbon turnover, are hotspots for deep-sea organic carbon degradation and unique microbial processes. However, little is known about the organic carbon components and microbial metabolisms driving their degradation in trench sediments. This study provides the first comprehensive quantification of total halogenated organic compounds (organohalides) in Mariana Trench sediments. The measured bulk organic halogen concentrations exceeded all previously reported individual compounds by orders of magnitude, with a mean stoichiometric ratio of 1:49 (halogen:carbon) in the sedimentary organic carbon pool. These findings suggest the trench sediments may represent a significant reservoir for organohalides. Metagenomic analysis of global ocean data shows significant enrichment of the genes for organohalides biodegradation (dehalogenation) in trench microbiomes than those in other marine environments. Putative dehalogenating microorganisms in trench sediments encompassed 16 phyla and 52 orders, capable of metabolizing 18 structurally diverse organohalide compounds, revealing an unexpectedly broad phylogenetic distribution of organohalides metabolism and versatile substrate specificity among trench microbial communities. High pressure microcosm experiments demonstrated rapid degradation of typical organohalide compounds and transcription of genes related to organohalides metabolisms, confirming an active organohalides degradation by trench microorganisms. These findings underscore the role of organohalides metabolism in organic carbon remineralization in hadal trenches, advancing our understanding of deep-sea carbon cycling and microbial survival.

## Introduction

The hadal trenches, which are found at ocean depths exceeding 6000 meters, are among the most inaccessible and least understood environments on the planet [1, 2]. Recent studies have shown that the hadal trench sediments contain significantly higher content of organic matter and exhibit much higher rates of microbial carbon turnover compare to other deep-sea environments, such as the abyssal plain [1, 3]. This has led to its recognition as hotspot for deep-sea organic carbon (OC) remineralization [1, 3]. Furthermore, the extreme conditions of the hadal zone, such as high pressure and geographic isolation, have led to the development of unique microbial communities and special metabolic processes, forming a distinct trench biosphere [4, 5]. Understanding the processes and mechanisms by which hadal microbes drive the degradation of sedimentary OC is essential for advancing knowledge of carbon cycling and life processes in deep-sea environments [1, 2].

Organic matter in deep-sea sediment are typically more recalcitrant than those from shallower depth, as the labile OC fractions have been largely consumed during the long-distance transportation from surface [6]. Biogeochemical evidence has shown accumulation of recalcitrant organic matter in hadal trench sediments, such as old and thermochemically stable fractions, humic-like fractions, as well as black carbon [7–9]. Correspondingly, microbial communities in trench sediments are dominated by taxa with functional traits to utilize persistent organic compounds, such as hydrocarbon and aromatic compounds [5, 10, 11], suggesting that the utilization of recalcitrant OC might be a crucial survival strategy for hadal microbes [10–12]. However, only selected metabolic processes (e.g. hydrocarbon degradation) have been experimentally validated [13, 14], leaving significant gaps in our understanding of OC transformation processes under extreme hadal trench conditions [2, 15, 16].

Halogenated organic compounds (HOCs), a group of recalcitrant organic compounds containing halogen elements, are generated in substantial quantities through various biotic and abiotic processes in natural environments [17, 18]. The ocean represents the largest source of natural HOCs on the planet [19]. In parallel, anthropogenic HOCs, recognized as environmentally hazardous substances and a major global concern, are widespread in the ocean [20]. The recalcitrant properties of HOCs facilitate their downward transport through water columns, leading to accumulation in deep-sea sediment reservoirs [20–22]. Recent studies have reported the presence of several HOC compounds in hadal trenches [23–28]. Moreover, metagenomic investigations have identified genes encoding HOC degradation pathways in dominant trench sediment taxa, suggesting potential to microbially metabolize these compounds [12, 29, 30]. These findings raise a pivotal question: Could microbial HOC degradation contribute meaningfully to OC remineralization in hadal sediments?

Addressing this question requires systematic investigation of (i) the content, composition, and storage of HOCs in the trench sediments; and (ii) prevalence, diversity, and metabolic activities of HOC-degrading microorganisms in these environments. However, current understanding remains severely limited. Only six studies have quantified HOCs in hadal trenches, exclusively focusing on four specific class of anthropogenic pollutants, including organochlorine pesticides, polychlorinated biphenyl (PCB), polybrominated diphenyl ethers (PBDE), and novel brominated flame retardants [23–28]. As the detected compounds showed low concentrations (pg/g scale) in trench sediments, the broader biogeochemical significance of HOCs has been overlooked. Similarly, current understanding on HOCs metabolism in trenches is restricted to genomic predictions from two bacterial taxa (i.e. *Chloroflexi* and *Pseudomonas*) [12, 29, 30]. It remains unclear whether the degradation of HOCs represents a common metabolic capability across hadal trench microbial communities. Furthermore, the specific degradation pathways, activities, and their broader implications for carbon cycling in these extreme environments are still poorly understood.

Here we present the first comprehensive analysis of the total HOCs in sediments of the Mariana Trench, revealing concentrations of bulk organic halogen orders of magnitude higher than previously measured pollutants. Metagenomic and genomic analysis further showed that genes encoding enzymes and pathways for HOCs degradation are significantly enriched in hadal trench sediments compared to other environments from the global ocean. More than half of the microbial taxa in the trench sediments were found to contain these genes, and microcosm experiments simulating trench conditions further confirmed active degradation of HOCs by trench microbial communities. These findings provide novel insights into the significance of HOCs and their microbial degradation processes in carbon cycling of the hadal zone, advancing our understanding of carbon dynamics and microbial survival strategies in Earth's deepest ecosystems.

## Material and methods

### Sampling sites and sample processing

**Figure 1:**
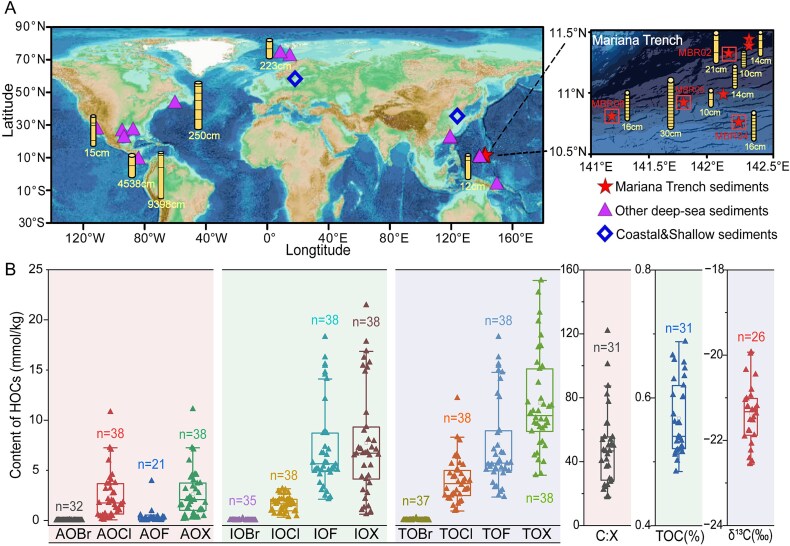
Sampling map and geochemical parameters. (A) Locations of sediment cores involved in the analysis of this study. Red stars enclosed in squares mark the sampling stations where detailed geochemical data were collected. The yellow columns with embedded black lines represent the subsampling layers of the sediment cores, whereas the numbers below denote the total core lengths. (B) Geochemical parameters of sediment cores from the Mariana Trench, including concentrations of AOH (AOF, AOCl, AOBr, and AOH), corresponding IOH (IOF, IOCl, IOBr, and IOH), and TOH contents (TOBr, TOCl, TOF, and TOH). Additionally, TOC, their carbon isotopic compositions (δ13C), and the calculated molar ratio of OC to halogen atoms (C:H) are presented.

Sediment samples from MBR02 (11.327°N,142.188°E, water depth 10 954 m)，MBR04 (10.761°N,142.274°E, water depth 5842 m), MBR05 (10.979°N, 141.950°E, water depth 6957 m), and MBR06 (10.813°N,141.180°E, water depth 6477 m) of the Mariana Trench were taken during the R/V *Tansuoyihao* TS15 cruise on December 2019 (Fig. 1). Samples were taken by a box corer attached to a Hadal Lander. The procedures of the sample processing were the same as previously described [12, 29]: after recovering onboard, the sediment samples were immediately subsampled using sterile polyamide corers, and most of the cores were stored under −80°C until analysis. The pore water was collected at 1–2 cm intervals using Rhizon samplers. Aliquots for DOM analysis were stored in a pre-combusted (525°C for 4 h) brown glass bottle, sealed with an acid-cleaned Teflon-lined cap, and frozen at −20°C.

### Measurement of halogenated organic matter and geochemical parameters

The method for sample pretreatment and HOCs measurement followed our previous study [31]. Briefly, sediment cores for HOCs measurement were thawed and divided at 2 cm interval along the vertical depth profile. The most outer layer of each depth fraction was removed to avoid halogen contamination, and the remaining sample was freeze-dried and ground. Twenty milligrams sediment were then weighted out from each sample and mixed with 50 ml sodium nitrate washing solution, shaking for 2 h at 140 rpm and then left overnight to remove inorganic halogens [31]. The sediment slurry was further processed to generate samples for measurement of insoluble and absorbable HOCs, and then subject to combustion and detection of halogen ions. Detailed procedures of sample processing, combustion, detection, and quality control can be found in supporting methodology.

Content (wt.%) and stable carbon isotopic composition of total organic carbon (TOC) were determined according to the previous reported procedures [32, 33], using an elemental analyzer connected to an isotope ratio mass spectrometer (Delta V Advantage, Thermo Scientific). Pore-water DOC concentrations were determined by a high-temperature catalytic combustion method using a Shimadzu TOC-L total carbon analyzer with a precision of ±3% [7]. Concentrations of inorganic nutrients (NO_3_^−^, NO_2_  ^−^, NH_4_  ^+^, and PO_4_  ^3−^) were determined using a QuAAtro autoanalyzer (Seal Analytical) [34], with a detection limit of 1 *μ*M and a precision of 2%. More details on measurement of biogeochemical parameters are provided in supporting methodology.

### Metagenomic sequencing and public data collection

Sediment cores were thawed on ice and were depth fractioned at 5 cm intervals. Total genomic DNA was extracted from 10–20 g of sediments from each depth fraction using the PowerMax® Soil Kit (MoBio Laboratories), and then purified by DNeasy® PowerClean Pro Cleanup Kit (QIAGEN). DNAs were fragmented to an average of 350 bp, and paired-end libraries were constructed and subjected to metagenomic sequencing on a MiSeq System (Illumina) in Majorbio Bio-Pharm Technology Co. Ltd (Shanghai, China). In total, eight metagenomes were successfully sequenced and generated 335.4 Gb of raw reads. The data size for individual metagenomes ranged from 27.2 to 50.1 Gb, with an average value of 41.9 Gb. The metagenomes were co-analyzed with 47 additional deep-sequenced metagenomes from the NCBI database to investigate the distribution of dehalogenase and associated microorganisms (Fig. 1, Table S1). The public datasets included 19 from Mariana Trench sediments, 27 from other global ocean habitats, and one enrichment culture from the Mariana Trench (MT6477m) (Table S1). The MT6477m dataset was used exclusively for genome retrieval to expand microbial diversity and was excluded from quantitative distribution analyses. The remaining publicly available metagenomes were selected to ensure broad geographic coverage (from shallow and marginal sites to deep-sea regions across multiple ocean basins), substantial bathymetric diversity (depth range from 72 m to 10 908 m), and sufficient sequence depth (>5 Gb), thereby ensuring analytical consistency.

### Assembly, functional annotation, and dehalogenase genes identification

Metagenomic reads from each sample were quality filtered using Trimmomatic v. 0.38 [35] with parameters specified as “LEADING:30 TRAILING:30 CROP:90 HEADCROP:10 SLIDINGWINDOW:4:25 MINLEN:50”, and were separately assembled with metaSPAdes v. 3.15.5 (−m 700) [36]. Gene predictions were performed against the assembled contigs using Prodigal v. 2.6.3 (−p meta) [37], and genes were then dereplicated to construct the non-redundant gene database using CD-HIT v. 4.8.1 with default parameters [38]. Functional annotation was performed using BlastKOALA against the KEGG database with default parameters [39]. The gene sequences annotated as any types of dehalogenases were extracted and confirmed using BLASTp search against UniProt protein database [40]. Only the sequences that showed ≥60% identity, ≥90% coverage, and E values <10^−5^ with existing dehalogenase sequences in the UniProt database were selected for downstream analysis.

### Relative abundance determination and phylogenetic analysis of dehalogenase

Clean reads from each metagenome were mapped back to confirmed dehalogenase genes sequences using BWA v. 0.7.17 [41]. BWA output was then converted to a sorted and indexed bam file using SAMtools v. 1.9 [42]. The number of mapped reads and length of each gene sequence from the bam file was utilized for calculation of the length normalized gene abundance, which was expressed as GPM (genes per million). The relative abundance of each type of dehalogenase was calculated by summing the GPM values of the confirmed gene sequences.

A total of 2300 BLASTp confirmed haloalkane dehalogenase gene sequences were extracted from the 27 Mariana Trench metagenomes, translated into protein sequences, and co-analyzed with 37 previously characterized haloalkane dehalogenase sequences retrieved from the Uniport database. The sequences were aligned with MAFFT v. 7.467 [43] and trimmed with trimAl software [44], both employing default parameters. A maximum-likelihood phylogenetic tree was constructed using IQ-Tree [45] with branch support evaluated based on 100 bootstrap replicates.

### Genome binning and dereplication

The quality-filtered reads were mapped back to the assembled contigs using Bowtie2 (v. 2.3.4.1) [46], and coverage was determined according to the mapping results with the jgi_summa- rize_bam_contig_depths script [46]. Metagenome binning was conducted for assemblies longer than 2500 bp using MetaBAT v. 2.12.1 [47] with default parameters. Quality of the metagenome-assembled-genomes (MAGs) was assessed by CheckM v. 1.1.2 using the line- age_wf workflow [48], and only MAGs with completeness >50% and contamination <5% were kept for further analysis. Redundant bins were subsequently dereplicated using dRep v. 2.3.2 [49] at 95% average nucleotide identity (ANI) (all other parameters were set to the default), and MAG with the highest quality was selected from each cluster for downstream analysis. The genome size was estimated by dividing the size of the MAG by its estimated completeness.

### Taxonomic assignment, identification of dehalogenating taxa and calculation of their relative abundance

Taxonomic classification of the MAGs was determined using GTDB-Tk, which is based on the phylogenetically calibrated Genome Taxonomy Database (GTDB, v.2.4.0) [50]. Coding sequences in the MAGs were predicted using Prodigal v. 2.6.3 with default setting [37]. Functional annotation was performed by using BlastKOALA against the KEGG database with default parameters [39]. The annotation results were manually examined to screen for the MAGs with dehalogenase annotated. The sequences of dehalogenase genes were then extracted from each candidate MAG and confirmed using BLASTp search against UniProt protein database. Only the MAGs containing gene sequences showing ≥60% amino acid identity and ≥90% coverage with existing dehalogenase in the UniProt database were selected as the potential dehalogenating microorganisms.

A phylogenomic tree was constructed for the confirmed dehalogenating MAGs, using the 43 universal single-copy genes (SCGs) used by CheckM [48]. Protein sequences of the SCGs were identified using HMMER v. 3.1b2 [51] with default parameters, individually aligned with MAFFT v. 7.467 [43], and then concatenated. Phylogenomic tree was constructed based on the alignment using FastTree2 v. 2.1.11 [52], with a JTT model, a gamma approximation, and 100 bootstrap replicates. Archaeal genome (*Halobacterium salinarum,* GCF_004799605.1) was utilized as the root. The phylogenomic tree was visualized with iTOL [53].

Relative abundances of the putative dehalogenating MAGs were calculated by mapping the trimmed reads to the manually curated and refined MAGs with CoverM (v.0.6.1; https://github.com/wwood/CoverM) in genome mode. The analysis included the dereplication flag using the default aligner Minimap2 (v.2.21; https://docs.csc.fi/apps/minimap2/) in short-read mode. The final relative abundance of each MAG is the percentage of the MAG in the mapped fraction of each sample.

### Reconstruction of metabolic network for putative dehalogenating microorganisms

The metabolic capabilities of the putative dehalogenating microbial taxa were explored by systematic annotation of MAGs containing dehalogenase genes. Coding sequences in the MAGs were annotated using the KEGG database [39], Cluster of Orthologous Groups (COG) [54] database, and PROKKA [55], following the steps in Liu *et al.* [12]. The pathways related with energy metabolism and biogeochemical cycling were also annotated with METABOLIC [56]. The metabolic network was reconstructed for each of the MAGs by summarizing the annotated pathways.

### High-pressure microcosms

#### Microcosm set up and sampling

Sediments were taken from site MBR06 (water depth 6477 m) of the Mariana Trench using a box corer during R/V *Tansuoyihao* TS15 cruise on December 2019. After removing the outer layers, around 2.0 kg sediment were taken by precombusted (450°C, 4 h) stainless steel scoops. Sediment samples were transferred into sterile incubation bags (polypropylene) and stored under 4°C for 20 months, which leads to the enrichment of putative dehalogenating microbial taxa (relative abundance of dehalogenating microbial taxa increased from 16.9% to 30.6% of total mapped metagenomic reads).

After thorough mixing with a precombusted stainless steel scoop, the sediments were divided into polypropylene incubation bags and mixed with 0.2 *μ*m filtered artificial seawater. Each of the 50 ml incubation bag contained 15 g sediment and 30 ml filtration sterilized seawater, and then either 1.5 mg of 4-Chlorobiphenyl (4-PCB) or 3 mg of γ-Hexachlorocyclohexane (γ-HCH) was amended as the representatives of anthropogenic HOCs. The selection of these substrates was guided by three criteria: structural distinction (aromatic vs. aliphatic), differing numbers of halogen atoms, and evidence of biodegradation pathways from trench sediment metagenomic annotations. No-substrate controls and no-microorganism controls were set up simultaneously with the experimental groups. No-substrate controls are incubation bags contained only sediment and sterilized seawater, and without HOCs substrates. No microorganism controls are incubation bags containing autoclaving sterilized sediments and filtration sterilized seawater, and either type of HOCs substrates with the same ratios as the experiment groups. All of the incubation bags for experimental groups and the controls were put into high pressure incubation vessels, and were incubated under 60 MPa and 4°C to simulate the *in situ* pressure and temperature. Triplicate incubation bags were taken for each treatment at day 0, day 30, day 60, day 90, and day 270 to monitoring the changes of organic substrates and microbial metabolisms.

#### 4-Chlorobiphenyl and γ-Hexachlorocyclohexane measurement

Around 15 ml of sediment slurry from each incubation bag was freeze dried and ground. After mixing with 100 *μ*L surrogate standard solution (2,4,5,6-Tetrachlorom-xylene, 500 mg/L), the sample was transferred into a Soxhlet extractor for extraction with acetone:n-hexane (1:1, v/v) (72°C, 20 h for 4-PCB, and 65°C, 22 h for HCH). The yielded extract from each sample was then evaporated to a final volume of 1 ml and then cleaned up in CNWBOND Florisil PR SPE cartridge (60–100 mesh, 1 g/6 ml). Before instrumental analysis, 100 *μ*L of the effluent was transferred into amber GC vials, and 50 *μ*L of injection standard (50 mg/L, 1-Bromo-2-nitrobenzene for 4-PCB analysis, Pentachloronitrobenzene for HCH analysis) was added to calculate recoveries. The 4-PCB and HCH were then analyzed by using gas chromatograph (Agilent 7890B). The detailed procedures on sample processing, instrument setting, as well as quality controls are provided in supporting methodology.

#### Microbial metabolic potentials and expression activities within microcosms

HOCs degradation related microorganisms and functional genes were studied via integrated metagenomic and metatranscriptomic analysis, according to the procedures provided in supporting methodology. Briefly, total DNA and RNA were coextracted from microcosm samples, and quality checked. DNA libraries were prepared directly, whereas RNA underwent amplification before library construction. Paired-end sequencing (150 bp) was performed on a NovaSeq 6000 System (Illumina) at Magigene Biotechnology Co. Ltd (Guangzhou, China). Quality filtered metagenomic reads were assembled, followed by gene prediction, and dereplication. HOCs degradation genes were identified via BLASTp. Relative abundance of dehalogenation related genes and genomes were quantified as GPM by read mapping. Relative abundances of the putative dehalogenating genomes were also calculated with CoverM (v.0.6.1, genome mode). Metatranscriptomic raw reads were quality filtered, and noncoding RNAs were removed. Clean reads were then mapped to the predicted protein coding genes from metagenome of the same sample. The transcription level of each gene or genome was expressed as transcripts per million (TPM).

## Results and discussion

### High content of organic halogens in hadal trench sediments

A method based on combustion-ion chromatography was developed to show high accuracy, precision, and reproducibility in determining the content of adsorbable organic halogens (AOH) including fluorine, chlorine, and bromine (AOF, AOCl, AOBr), and the corresponding insoluble organic halogens (IOH, including IOF, IOCl, IOBr), as well as total organic halogen contents (TOH, including TOBr, TOCl, TOF), in marine sediments [31]. In this study, the content and composition of organic halogens were determined in 38 sediment samples of four sediment cores taken along a depth gradient (5842 to 10 954 meters below sea surface) in the Mariana Trench, which resulted in a total of 430 measurements (Fig. 1; Fig. S1; Table S2). Organic halogens were detected in all of the trench sediments tested (Fig. 1B). The values of AOH, which indicates dissolved organic halogens adsorbable by activated carbon, ranged from 0.14 ± 0.02 to 11.10 ± 1.81 mmol/kg (dry weight of sediment) (Fig. 1B). The insoluble HOCs (IOH) ranged from 0.62 ± 0.19 to 21.46 ± 0.59 mmol/kg (Fig. 1B), which were significantly higher than the content of AOH (*P* < .01, t-test). The dissolved fraction of organic halogens was mainly dominated by organic chlorine compounds (AOCl), which were significantly more abundant than AOBr and AOF (*P* < .01, t-test). In contrast, organic fluorine compounds (IOF) showed the highest abundance in the IOH, followed by IOCl (Fig. 1B). Organic bromine compounds showed the lowest abundance in both the dissolved and insoluble HOCs (Fig. 1B).

The concentration of TOH in sediments of the Mariana Trench ranged from 4.51 ± 0.02 to 23.93 ± 0.67 mmol/kg (mean: 11.69 mmol/kg), with organic chlorine constituting the predominant fraction (Fig. 1B). TOC content remained relatively stable across samples (0.48%–0.69%, mean 0.57 ± 0.06%), yielding carbon to halogen molar ratios (C:H) of 17.8 to 121.8 (mean 49.2) (Fig. 1B). This corresponds to approximately one halogen atom for every 49 OC atoms. Comparative analysis revealed significantly higher TOH concentrations and lower C:H ratios in trench sediments relative to coastal and shelf sediments (*P* < .05, t-test) (Fig. S2) [31]. In addition, the average Cl: C ratio (1:168) in trench sediments far exceeded that of deep-sea particles (1:500) (Leri *et al.* 2015 [21]). These results indicate accumulation of organic halogens in sedimentary OC pool of the Mariana Trench. However, broader validation through comprehensive TOH measurements across global ocean is required to confirm these patterns.

Although anthropogenic HOCs, such as PCBs, PBDEs, and halogenated organic pesticides have been documented in trench ecosystems [23, 24, 26, 28], these compounds generally showed low concentration in sediments (up to 4.2 *μ*g/kg) [57]. Our measurements reveal TOH concentrations (average 292.47 mg/kg) orders of magnitude higher than those known pollutants (Table S3). This disparity suggests that the Mariana Trench sediments harbor a vast, uncharacterized diversity of HOCs beyond currently identified compounds. Multiple sources may contribute to this diverse HOC pool, including anthropogenic inputs and their transformation products [23, 24, 26, 28], biological production through marine organism metabolism, and abiotic formation via physico-chemical halogenation of natural organic matter [18, 19, 21]. This chemical diversity is further supported by Cai *et al.* [58], who identified as many as 965 distinct halogenated organic molecules from dissolved organic matter (DOM) of natural seawater, demonstrating that HOCs constitute a major component of previously uncharacterized DOM (dark DOM) that significantly contributes to marine recalcitrant carbon pools. Although the present study does not provide structural identification of individual HOCs in trench sediments, the abundant organic halogens measured strongly suggests their quantitative importance in shaping the recalcitrant OC reservoir in the deepest oceanic ecosystem.

### Enrichment of dehalogenases in sediments of the Mariana Trench

**Figure 2:**
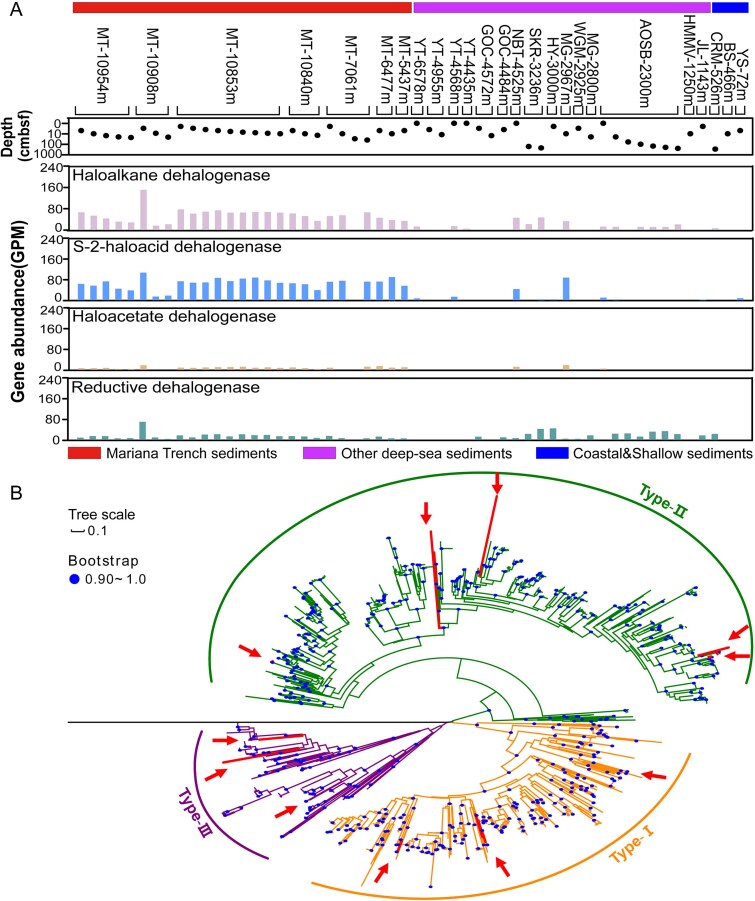
Distribution and phylogenetic analysis of dehalogenases. (A) Relative abundance of different types of dehalogenases in the metagenomes from the global ocean. The values were expressed as GPM. (B) Maximum-likelihood phylogenetic tree of haloalkane dehalogenase identified from the Mariana Trench sediment. Red arrows indicate the location of reference haloalkane dehalogenase sequences from the Uniport database. Epoxide hydrolase (accession no. P07099) served as the root.

The potential for trench microorganisms to metabolize HOCs was investigated through metagenomic analysis on occurrence, relative abundance, and distribution of dehalogenase, which is the key enzyme for the biodegradation of HOCs [59–61]. A total of 54 metagenomes from global ocean sediments were examined, comprising 27 deeply sequenced metagenomes from sediment cores collected from the Mariana Trench, alongside 27 metagenomes from other deep-sea and shallow/coastal sediments (Fig. 2A, Table S1). The results indicated the presence of dehalogenases in 26 of the 27 metagenomes derived from Mariana Trench sediments (Fig. 2A). Specifically, four distinct types of dehalogenases were identified: haloalkane dehalogenase, S-2-haloacid dehalogenase, haloacetate dehalogenase, and reductive dehalogenase (Fig. 2A). These enzymes facilitate the biodegradation of HOCs through either hydrolytic or reductive mechanisms [19], indicating a significant metabolic versatility towards HOCs utilization within trench sediments. The relative abundance of haloalkane dehalogenase and S-2-haloacid dehalogenase was significantly higher in Mariana Trench sediments compared to other deep-sea and shallow sediments (*P* < .001, t-test) (Fig. 2A), indicating an enrichment of dehalogenation metabolism within the microbiome of the Mariana Trench. These results align well with the elevated concentrations of organic halogens detected in the sediment of the trench (Fig. 1B, Fig. S2), implying that trench microorganisms may utilize HOCs as substrates for living.

Phylogenetic analysis of haloalkane dehalogenase sequences from the Mariana Trench metagenomes revealed that the microbial communities in the Mariana Trench encompass all three types of haloalkane dehalogenases (HLD-I, II, and III) (Fig. 2B). As different types of the enzyme exhibit varying substrate specificities [62], and co-occurrence of different enzymes would greatly expand the substrate spectrum of the trench microbial community, thereby enhance the overall efficiency of HOCs metabolism.

### Prevalence of dehalogenation metabolism among hadal trench microorganisms

**Figure 3:**
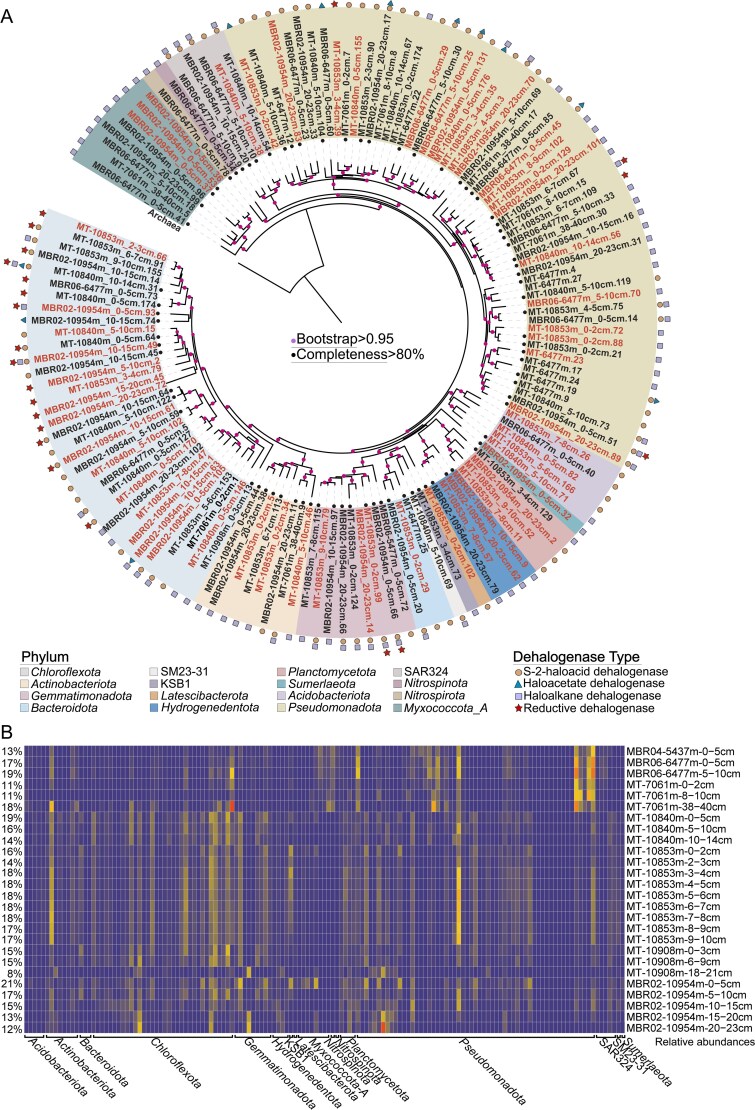
Phylogeny and distribution of microbial taxa with dehalogenation potentials. (A) Maximum-likelihood phylogenomic tree of the 143 MAGs harboring genes encoding dehalogenase. The tree was rooted using an archaeal genome (*Halobacterium salinarum*, accession no. GCF_004799605.1). Phylum level taxonomy is represented by colored backgrounds, whereas different symbols indicate the types dehalogenase genes identified in each MAG. Red colored text indicate the novel species. (B) Relative abundance of these MAGs across sediment samples from the Mariana Trench. The cumulative relative abundance of the 143 MAGs is indicated numerically on the left.

A total of 2544 MAGs were reconstructed from the 27 Mariana Trench sediment metagenomes. Dereplication at 95% ANI yielded 285 representative genomes, of which 50.2% (143 MAGs) harbored dehalogenase genes (Fig. 3A; Table S4). These MAGs (putative dehalogenating MAGs) showed contamination <5.00%, the genome size ranged from 0.58 to 5.31 Mbp, the GC content ranged from 59.4% to 65.4%, and 79 of them had a genome completeness >80% (Fig. 3A; Table S4). Among the 143 putative dehalogenating MAGs, 78 MAGs contained genes for S-2-haloacid dehalogenase, 11 for haloacetate dehalogenase, 79 for haloalkane dehalogenase, and 23 for reductive dehalogenase. Although the majority of the MAGs harbored a single type of dehalogenase, 40 MAGs contained genes for multiple dehalogenase types, with one MAG from the *Chloroflexota* phylum (MT_10840m_10–14 cm.31) possessing genes for all four dehalogenases (Fig. 3A).

Phylogenomic analysis (GTDB database, v. 2.4.0) revealed that the 143 putative dehalogenating MAGs were distributed across 16 bacterial phyla and 20 classes, including 60 novel species and 15 novel genera (Fig. 3A; Table S4). By comparing against the mibPOPdb database [63, 64], our analysis revealed dehalogenation potential in ten bacterial phyla previously not known to harbor this capability (Fig. 3A; Table S4). Thus, the findings offer evidence for a greater diversity of dehalogenating microorganisms than previously appreciated. The putative dehalogenating MAGs were found to be widely distributed across all analyzed sediment samples, representing 8% to 21% of metagenome reads (Fig. 3B). The majority of the MAGs (96 MAGs) showed high occurrence frequency, being present in over 80% of the sediment samples analyzed (Fig. 3B, Table S5). These findings highlight the notable abundance and high prevalence of putative dehalogenating microorganisms within the microbial communities of Mariana Trench sediments.

**Figure 4:**
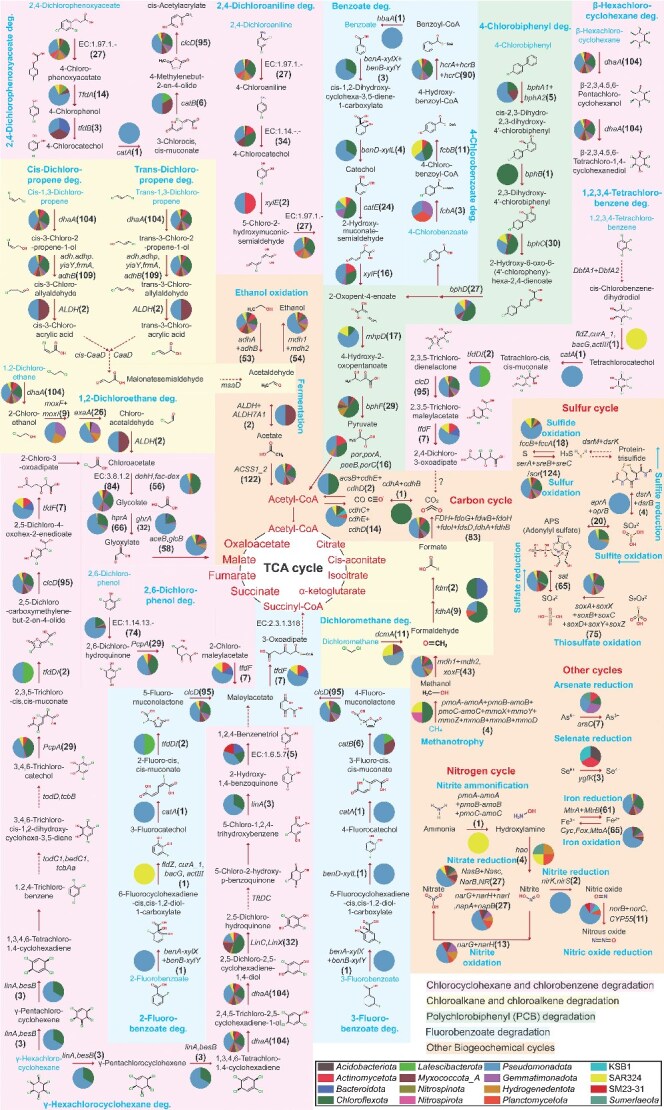
Metabolic pathways of the microbial taxa with dehalogenation potentials. Comprehensive degradation pathways of HOCs mediated by dehalogenation microbial consortia (the 143 MAGs) retrieved from the Mariana Trench sediments, and their associated biogeochemical cycling processes were illustrated. The name of the encoding gene involved in each step of degradation was listed, and the value in the brackets shows the number of MAGs containing the gene. The pie charts beside the gene names indicate the phylum level taxonomic distribution and proportional representation of MAGs containing each gene.

Functional annotation suggested that the putative dehalogenating population (143 potential dehalogenating MAGs) contained gene sets that encode pathways for the degradation of 18 HOCs compounds with distinct molecular structures, categorized as groups of chlorocyclohexane and chlorobenzene, chloroalkane and chloroalkene, fluorobenzoate, as well as polychlorobiphenyl (PCB) (Fig. 4, Fig. S3, Table S6–S8). These represent nearly all documented degradation pathways for HOCs in the KEGG database, suggesting a wide range of substrate spectra for the putative dehalogenating population identified. Among the 18 annotated compounds, 15 were predicted to be completely degraded via entering the TCA cycle (Fig. 4, Table S6–S8). Furthermore, genes encoding each functional enzyme in these pathways were distributed across multiple bacterial lineages (Fig. 4, Table S6), indicating a functional redundancy that could facilitate the efficient degradation of substrates [65]. The exact molecular composition of HOCs present in trench sediment remains undetermined, but likely comprise a mixture of both anthropogenic and natural sources. Anthropogenic HOCs showed low structural diversity [17], with majority of their degradation pathways being annotated in our analysis (Fig. 4, Table S6–S8). In contrast, natural HOCs exhibit a considerably higher structural diversity [17, 18]. Yet, phytoplankton and sinking particles, presumably the main organic matter sources in hadal trenches, contain primarily aliphatic HOCs [21]. Considering the diverse aliphatic HOCs degradation pathways identified in the MAGs (Fig. 4), the putative dehalogenating population possess the potential to efficiently utilize HOC substrates present in hadal trenches.

In addition to direct degradation of HOCs, the putative dehalogenating microorganisms exhibited potentials to contribute to other ecological functions. Firstly, the annotated degradation pathways of HOCs involved many enzymatic steps, which produce a series of intermediates (Fig. 4). These intermediate compounds are generally more bioavailable than the original HOCs, and can be utilized by other heterotrophic taxa, thereby positioning putative dehalogenating microorganisms as crucial ecological mediators that facilitate microbial interactions in organic matter remineralization within trench sediments [17, 66, 67]. In addition, the putative dehalogenating microbial populations also showed the capability to drive the biogeochemical cycling of multiple key elements other than halogen and carbon. Particularly, sulfur oxidation seemed to be common within the putative dehalogenating population, with 49%–87% of the MAGs harboring gene sets for different steps of sulfur oxidation (Fig. 4, Fig. S3, Table S6–S8). Moreover, 43% and 45% of the MAGs also showed potentials of iron reduction and iron oxidation, respectively (Fig. 4, Fig. S3, Table S6–S8). The findings demonstrate a significant role of putative dehalogenating microorganisms in the functions and structures of hadal trench ecosystems.

### Active halogenated organic compounds degradation driven by hadal trench microorganisms

**Figure 5:**
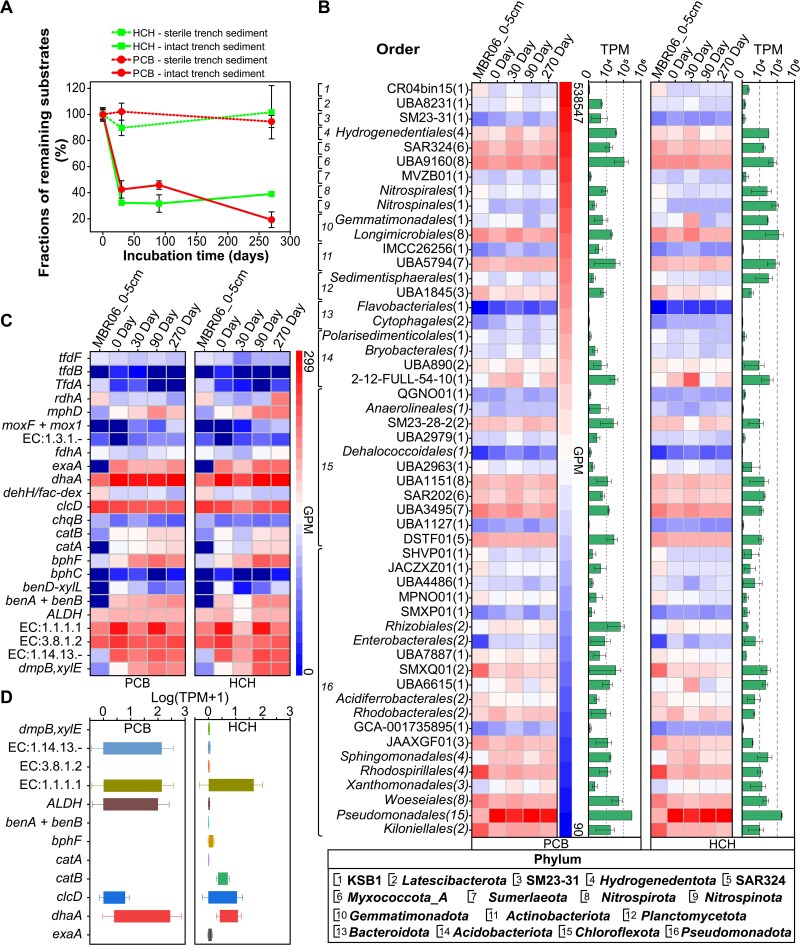
Microbial degradation of typical HOCs compounds by sediment communities from the Mariana Trench, under simulated high-pressure microcosm conditions. (A) Temporal decrease in substrate concentrations over incubation time in treatments with intact trench sediments. (B) Dynamics of relative abundance (GPM) and transcriptional activity (TPM) of MAGs harboring dehalogenase genes at different incubation time points. (C) and (D), Temporal changes in relative abundance and transcriptional activity of functional genes associated with HOCs degradation pathways.

The degradation activity of HOCs by hadal trench microorganisms was demonstrated by inoculating typical HOCs compounds—4-PCB and γ-HCH—into sediments from the Mariana Trench (MBR06, 6477 m depth), and incubating them under high pressure (60 MPa) and low temperature (4°C) to simulate *in situ* conditions. Time-series sampling and analysis revealed a rapid decrease of substrate concentration in all treatments containing trench microorganisms (nonsterile systems), whereas no significant changes occurred in the control systems (sterile sediments) (Fig. 5A). For both substrates, the most substantial decrease took place within the first 30 days, leaving <48% of the original concentration. PCB levels continued to decline throughout the 270-day period, whereas γ-HCH remained relatively stable after Day 30 (Fig. 5A).

Metagenomes and metatranscriptomic analyses indicated that the 143 putative dehalogenating MAGs identified in trench sediments were present and actively expressed in the microcosms (Fig. 5B, Table S9). Dominant MAGs in both metagenomes and metatranscriptomes were affiliated with the orders UBA1845 (phylum *Planctomycetota*); *Hydrogenedentiales* (*Hydrogenedentota*); SAR324 (SAR 324); *Longimicrobiales* (*Gemmatimonadota*); UBA5794 (*Actinomycetota*); UBA9160 (*Myxococcota*); *Woeseiales*, *Pseudomonadales*, and *Kiloniellales* (*Pseudomonadota*); as well as SM23-28-2, UBA1151, SAR202, UBA3496, and DSTF01 (phylum *Chloroflexota*) (Fig. 5B). Among these, MAGs from the order *Pseudomonadales* were the most abundant in the microcosms (Fig. 5B), underscoring their potential role in HOC degradation.

Comparing with no substrate control, the majority of functional genes associated with HOC degradation pathways showed elevated relative abundance in the metagenomes of the microcosms amended with substrates (t-test; *P* < .05 for seven genes; 0.05 < *P* < .10 for another seven genes; Fig. 5C, Table S10). Over half of these genes being actively expressed in transcriptomes, which were retrieved from samples of Day 90 (Fig. 5D, Table S10). Key functional genes, including *dhaA* (encoding haloalkane dehalogenase), *EC:1.1.1.1* (alcohol dehydrogenase), *clcD* (carboxymethylenebutenolidase), *catB* (muconate cycloisomerase), *EC1.14.13.-* (2-octaprenylphenol hydroxylase), and *ALDH* (aldehyde dehydrogenase), exhibited elevated transcriptional activity compared to other genes involved in HOCs degradation pathways (Fig. 5D). This highlights their important roles in these metabolic processes. The absence of certain pathway genes (e.g. *linA*, *bph*; Fig. 5, Table S10) in both metagenomic and metatranscriptomic datasets suggests the potential involvement of novel enzymes in the initial breakdown of γ-HCH and PCB within microbial communities in the microcosms. Additionally, the lack of detectable expression for some enzymes in the transcriptomic data may also be attributed to the sampling time point (Day 90), which corresponds to the middle stage of the degradation process, during which relevant gene expression may have already subsided. We also acknowledge that the absence of successful metatranscriptomic sequencing for the no-substrate controls limits the ability to definitively attribute all transcriptional changes solely to substrate amendment. Nevertheless, the consistent enrichment of other degradation genes in the metagenome, coupled with their concurrent activation in the transcriptome, provides supportive multi-layered evidence points to the substrate-induced microbial activity.

Currently, experimental studies on microbial degradation of HOCs were primarily conducted in coastal or shallow offshore environments [68]. Although a handful of investigations have reported HOCs degradation activities in deep-sea microorganisms [69–72], these assessments were mainly performed under laboratory conditions (15°C–30°C at atmospheric pressure). Our work extends these investigations by characterizing both degradation kinetics and functional gene expression under stimulated *in situ* hadal trench conditions. Collectively, the integrated chemical and microbial analyses from the microcosm experiments demonstrated rapid reduction of HOCs substrates, alongside the active expression of dehalogenation-associated microbial populations and functional genes. These findings demonstrate the capacity of hadal trench microbial communities to actively metabolize HOCs under the simulated *in situ* high pressure and low temperature conditions. The two tested compounds, 4-PCB (aromatic, mono-chlorinated) and γ-HCH (aliphatic, hexa-chlorinated), represent HOC molecules with distinct structures and halogenation levels. The degradation activities observed are therefore well align with the broad substrate spectra predicted by genomic reconstructions of hadal microbial metabolic networks (Fig. 4). However, this study was limited to two anthropogenic HOCs, further research is necessary to explore the metabolic activity of deep-sea microorganisms on other HOCs compounds, particularly those of natural origin. Additionally, as this experiment utilized enriched sediment microorganisms, the degradation rates observed may deviate from those in actual trench environments. Future *in situ* cultivation studies are needed for a more accurate evaluation of the rate and flux of HOCs degradation in hadal trench ecosystems.

### Implications for deep-sea biogeochemical cycling and environmental bioremediation

The ecological significance of microbial dehalogenation processes within deep-sea carbon cycling remains poorly understood. Our study advances the current understanding through three key discoveries: (i) quantification of TOHs in trench sediments at concentrations exceeding reported HOC compounds by orders of magnitude, revealing a previously underestimated organic halogen pool, (ii) metagenomic evidence demonstrating the enrichment of functional genes for HOCs metabolism in trench sediments, and their widespread distribution across 16 phyla of trench microbes, and (iii) experimental verification of active degradation of HOCs with different molecular structures under simulated trench conditions (Table S11). These findings suggest that HOCs likely serve as important substrates for microorganisms inhabiting the Mariana Trench, and that the metabolism of HOCs may represent a common strategy for the survival of microorganisms in trench environments. Our results further implicate microbial HOCs degradation as a potentially important component of carbon cycling in the Mariana Trench. In addition to the Mariana Trench, our global metagenomic results demonstrated that dehalogenase genes present in microbial metagenomes from various habitats across the global ocean (Fig. 2A), with potential dehalogenating microorganisms, such as *Chloroflexi*, being widespread and dominant in hadal trench sediments and other deep ocean habitats [72–74]. Dehalogenation activities have even been recorded in deep subseafloor sediments at depth up to 358 meters below the seafloor [70, 71]. These evidences collectively suggest that HOC metabolism is likely ubiquitous in deep-sea environments, indicating that microbial degradation of HOCs may play a crucial yet overlooked role in the global carbon cycle, warranting further investigation in future studies.

Although anthropogenic HOCs are typically considered hazardous environmental pollutants that ultimately accumulate in deep-sea sediments [20, 22], our results demonstrate that HOCs can serve as substrates for a diverse array of microbial taxa in deep-sea environments, where they can be effectively degraded. This provides valuable new insights into the fate and environmental implications of HOC pollutants within deep-sea ecosystems. Moreover, the high diversity of putative dehalogenating microorganisms, along with the diverse dehalogenase identified in hadal trenches, represents promising resources for environmental bioremediation applications.

## Supplementary Material

Supporting_methodology_and_figures_wraf273

Table_S1-S11_wraf273

## Data Availability

Sequences of the metagenomes from the field sediment samples and microcosm experiments are available in the NCBI database with the project number PRJNA1236873. The sequences of metagenome-assembled-genomes (MAGs) can be accessed under PRJNA1236908. Sequences metatranscriptoms recovered from the simulated high-pressure microcosms are available under the accession number PRJNA1236900.
