# Supplementary material for: Extensive halogenated organic compound reservoirs and active microbial dehalogenation in Mariana Trench sediments
Source: ISME J. 2025 Dec 10;19(1):wraf273. doi: 10.1093/ismejo/wraf273 (PMC12750322; doi:10.1093/ismejo/wraf273)
Supplement: Supporting_methodology_and_figures_wraf273 [file supporting_methodology_and_figures_wraf273.pdf]

Supplementary Materials

Extensive halogenated organic compound reservoirs and active microbial dehalogenation in Mariana Trench sediments

Short title: Trench microbes degrade organohalides

Rulong Liu[1#*], Hui Wei[1#], Zhiao Xu[1#], Yuheng Liu[1], Jiani He[1], Zhixuan Wang[1], Li Wang[1*], Min Luo[1], Jiasong Fang[1, 2*], Federico Baltar[1, 3], Yunping Xu[1], Qirui Liang[1], Liting Huang[1]

[1], College of Oceanography and Ecological Science, Shanghai Ocean University, Shanghai, China

[2] Laboratory for Marine Biology and Biotechnology, Qingdao National Laboratory for Marine Science and Technology, Qingdao, China

[3] Department of Functional and Evolutionary Ecology, University of Vienna, Vienna, Austria

***Correspondence:**

Rulong Liu, College of Oceanography and Ecological Science, Shanghai Ocean University, 999 Huchenghuan Road, Lingang, Pudong, Shanghai 201306, China. E-mail: rlliu@shou.edu.cn;

Li Wang, College of Oceanography and Ecological Science, Shanghai Ocean

University, 999 Huchenghuan Road, Lingang, Pudong, Shanghai 201306, China. E-mail: wang-l@shou.edu.cn;

Jiasong Fang, College of Oceanography and Ecological Science, Shanghai Ocean University, 999 Huchenghuan Road, Lingang, Pudong, Shanghai 201306, China. E-mail: jsfang@shou.edu.cn.

[#], these authors contribute equally.

Supporting Methodology

Measurement of halogenated organic matter and geochemical parameters

The method for sample pretreatment and HOCs measurement followed our previous study [1]. Briefly, sediment cores for HOCs measurement were thawed and divided at 2 cm interval along the vertical depth profile. The most outer layer of each depth fraction was removed to avoid halogen contamination, and the remaining sample was freeze-dried and ground. Twenty milligrams sediment were then weighted out from each sample and mixed with 50 mL sodium nitrate washing solution, shaking for 2 h at 140 rpm and then left overnight to remove inorganic halogens [1]. The sediment slurry was then filtered through a polycarbonate (PC) membrane to collect insoluble fraction of the sediment. Eighty milligrams of activated carbon were added to the filtrate and shake for 2 h at 140 rpm. The activated carbon with dissolved organic matter adsorbed was collected on a PC membrane by filtration. The two PC membranes were then subject to combustion for measurement of content of insoluble and absorbable HOCs, respectively. The protocols for combustion and detection of different halogen ions by IC were the same as Wei et al. [1]. All labware utilized were carefully selected and pretreated according to our previous study to secure that they were free of halogen contamination [1]. Three replicates were taken for each sample. Triplicate sample processing blanks and analytical blanks were involved in each batch of experiment to indicate the presence and level of the background contamination. All measured values were calibrated by subducting the background values in blank controls from the same batch of experiment.

Content (wt.%) and stable carbon isotopic composition of TOC were determined according to the previous reported procedures [2,3], using an elemental analyzer

connected to an isotope ratio mass spectrometer (Delta V Advantage, Thermo Scientific). Prior to analysis, sediment samples were acidified with 1 M HCl to remove inorganic carbon. Stable carbon isotope results were reported using the per mil notation (δ, ‰) relative to the V-PDB standard. The average standard deviation of each measurement, determined by replicate analysis, was ± 0.03% for TOC and ± 0.2‰ for $\delta^{13}C$. Pore-water DOC concentrations were determined by a high-temperature catalytic combustion method using a Shimadzu TOC-L total carbon analyzer with a precision of ± 3% [4]. Concentrations of inorganic nutrients (NO_3^-, NO_2^-, NH_4^+, and PO_4^{3-}) were determined using a QuAAtro autoanalyzer (Seal Analytical) [5], with a detection limit of 1 μM and a precision of 2%.

4-PCB and γ-HCH measurement in microcosm samples

Around 15 mL of sediment slurry from each incubation bag was freeze dried and ground. After mixing with 100 μL surrogate standard solution (2,4,5,6-Tetrachlorom-xylene, 500 mg/L), the sample was transferred into a Soxhlet extractor for extraction with acetone:n-hexane (1:1, v/v) (72 °C, 20 h for 4-PCB, and 65 °C, 22 h for HCH). The yielded extract from each sample was then evaporated to a final volume of 1 mL and then cleaned up in CNWBOND Florisil PR SPE cartridge (60-100 mesh, 1 g/6 mL). First, 1 g of Na_2SO_4 was added to the cartridge and 10 mL of n-hexane was added to pre-condition the column. Then the extract was transferred into the column and eluted by 10 mL of acetone:n-hexane (1:1). The collected effluent was concentrated to 5 mL. Before instrumental analysis, 100 μL of the effluent was transferred into amber GC vials, and 50 μL of injection standard (50 mg /L, 1-Bromo-2-nitrobenzene for 4-PCB analysis, Pentachloronitrobenzene for HCH analysis) was added to calculate recoveries.

The 4-PCB and HCH were analyzed by using gas chromatograph (Agilent 7890B). A HP-5MS column (30 m× 0.25 mm×0.25 μm) and a mass selective detector (MSD) were utilized for analysis of 4-PCB, while a DB-5 column (15 m× 0.32 mm×0.25 μm) and a 63Ni-ECD detector were utilized for analysis of HCH. Samples (1 μL) were injected in the splitless injection mode with high purity helium gas (1 mL/min) as carrier gas for 4-PCB analysis, and nitrogen gas (2 mL/min) for HCH analysis. For analysis of 4-PCB, the temperature of the column was set at 40 °C for 2 min, then raised to 280°C (20 $°C·min^{-1}$), and finally retained at 280 °C for 5 min. The injection port and detector temperatures were kept at 270 °C and 280 °C, respectively. Electron ionization energy was set at 70 eV, and ion source and quadruple temperatures were kept at 230 ° and 150 °C, respectively. For analysis of HCH, the temperature of the column was set at 100 °C for 2 min, raised to 220 °C (15 $°C·min^{-1}$) and retained for 5 min, then raised to 260 °C (15 $°C·min^{-1}$) and retained for 20 min. The injection port and detector temperatures were kept at 220 °C and 300 °C, respectively.

The quality control steps included sample processing blanks, limit of detection, recovery tests, replicates, and standard curves. Sample processing blanks were involved for each batch of experiment to indicate possible contamination. All blanks were below the limits of detection (LOD). The LODs were set to be triple the standard deviation of the blanks, which were averagely 0.1 μg/g for PCB and 0.01 μg/g for HCH. Spiked recovery tests of the standards showed acceptable recoveries ranging from 81.80% to 85.40% for 4-PCB, with the relative standard deviation (RSD) less than ± 2.57%, and from 91.59% to 94.63% for HCH, with the RSD less than ± 4.74%. The standard curves had high degree of linearity ($R^2 > 0.999$) for all the compounds.

Metagenomic and metatranscriptomic analysis for microcosm samples

Total DNA and RNA were coextracted from each of the microcosm samples using PowerSoil Total RNA Isolation Kit and DNA Elution Accessory Kit (MoBio Lab, United States). The concentration and quality of DNA and RNA were determined using both Qubit 4.0 (Thermo Fisher Scientific, Waltham, USA) and Nanodrop One (Thermo Fisher Scientific, Waltham, USA). Quality of RNAs were also determined through the Agilent 4200 system (Agilent Technologies, Waldbronn, Germany). All of the DNA samples were directly subject to library preparation using the ALFA-SEQ DNA library Prep Kit (Findrop Biosafety Tech., China). The RNA samples went through whole transcriptome amplification using RNA REPLI-g Cell WGA & WTA Kit (QIAGEN, Germany), and subject to library construction using ALFA-SEQ RNA library Prep Kit II (Findrop Biosafety Tech., China). The DNA libraries and RNA libraries were then subject to metagenome and metatranscriptome sequencing, respectively, in Magigene Biotechnology Co. Ltd (Guangzhou, China) using Illumina novaseq6000 (PE150 sequencing).

Metagenomic reads from each sample were quality filtered, assembled, and genes were predicted and dereplicated following the same procedure as metagenomic analysis from field samples. Functional annotation was performed against the KEGG, COG and PROKKA databases. The gene sequences involved in any of the HOCs degradation pathways were extracted and confirmed via BLASTP. Clean reads from each metagenome were mapped back to predicted genes using BWA v. 0.7.17 and the gene length normalized relative abundance of each gene were expressed as GPM. The relative abundances of the dehalogenation genomes were estimated by mapping the metagenomic reads to sequences of the corresponding MAGs and expressed as GPM. Relative abundances of the dehalogenation MAGs were also calculated with CoverM

(v.0.6.1; https://github.com/wwood/CoverM) under the genome mode, and expressed as percentage of the total mapped reads).

Duplicated metatranscriptomes were successfully sequenced for both PCB and HCH microcosm samples taken from 90th day. Metatranscriptomic raw reads were quality filtered using Trimmomatic v. 0.38 [6], and non-coding RNAs were removed using sortmerna v. 4.2.0 [7]. Clean reads of each metatranscriptome were then mapped to the predicted protein coding genes of the metagenome from the same sample. The transcription level of each gene was expressed as transcripts per million (TPM). Similarly, the transcription level of the dehalogenation genomes was estimated by mapping the metatranscriptome clean reads to sequences of the corresponding MAGs and expressed as TPM.

Supporting Figures

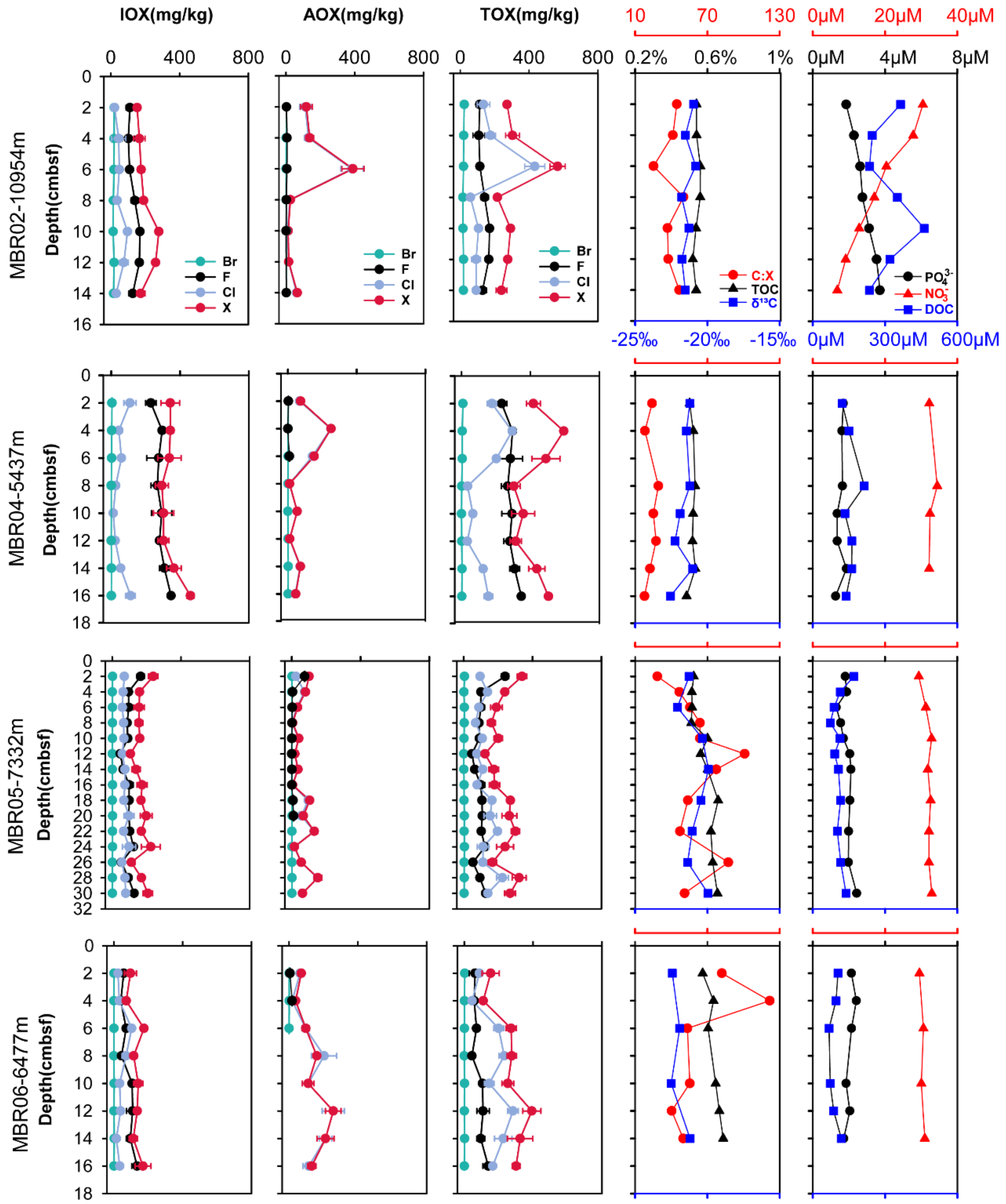

Figure S1. Concentration and distribution of TOX and geochemical parameters.

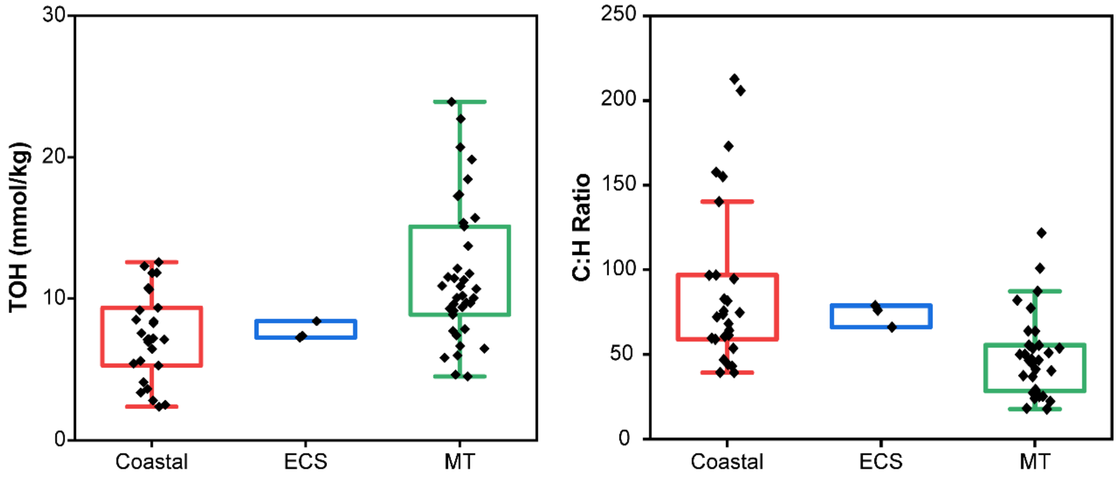

Figure S2. Comparison of TOH values and C:H ratio between the sediments of the MT and those from other deep-sea or coastal area. The data of coastal and Eastern China Sea (ECS, water depth 950 m) was retrieved from Wei et al. [1].

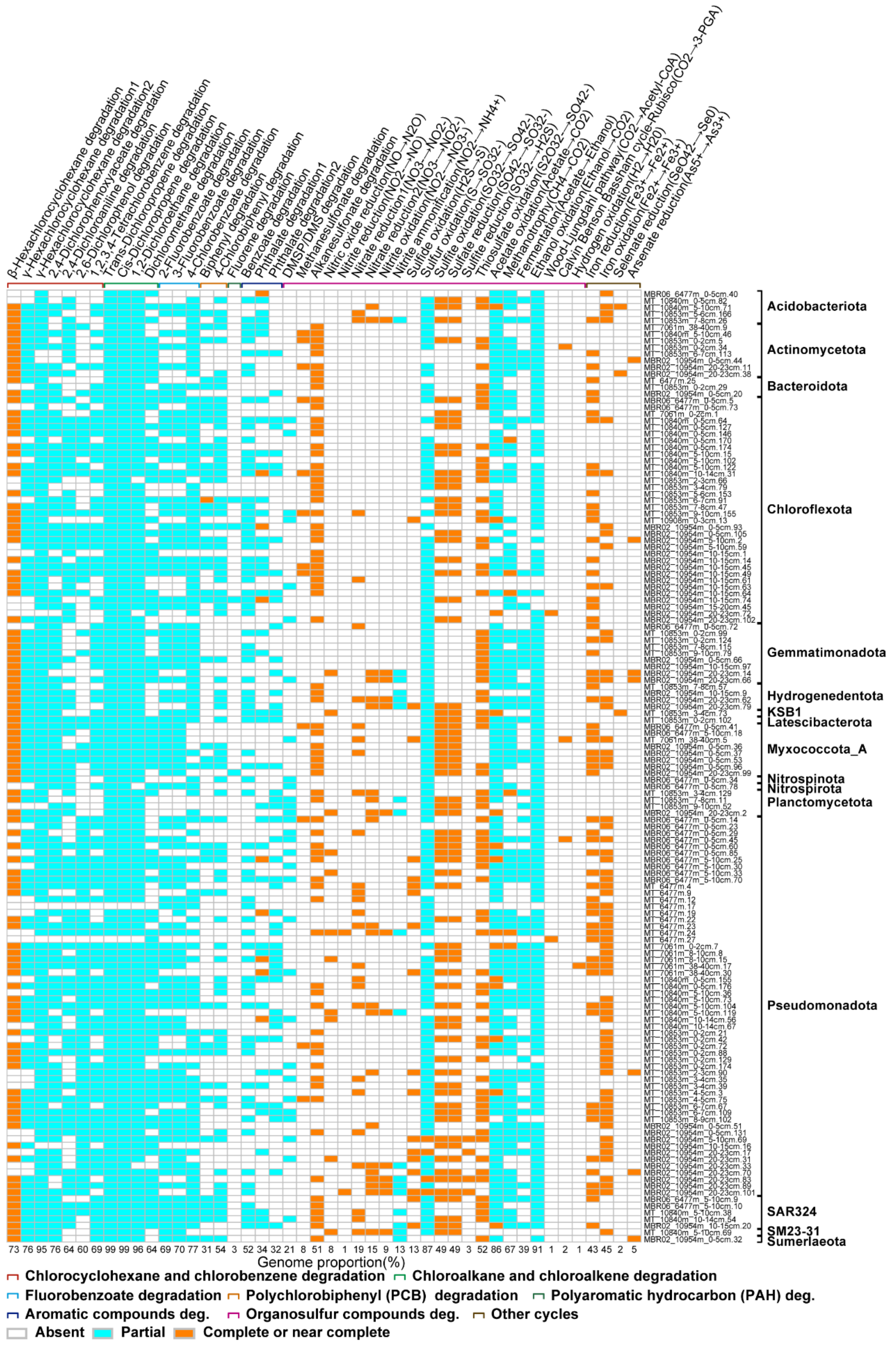

Figure S3. Presence and completeness of metabolic pathways in each of the MAGs with dehalogenation potentials. Detailed information can be found in Table S7 and Table S8.

References:

1. Wei H, Liu YH, Huang LT, et al. Determining the abundance, composition and spatial distribution of organohalogens in marine sediments using combustion-ion chromatography. *Mar Environ Res* 2024;**199**:106626.
2. Luo M, Glud RN, Pan BB, et al. Benthic Carbon Mineralization in Hadal Trenches: Insights From In Situ Determination of Benthic Oxygen Consumption. *Geophys Res Lett* 2018;**45**:2752-2760.
3. Xu YP, Li XX, Luo M, et al. Distribution, Source, and Burial of Sedimentary Organic Carbon in Kermadec and Atacama Trenches. *J Geophys Res Biogeosci* 2021;**126**: e2020JG006189.
4. Hu TC, Luo M, Wünsch UJ, et al. Probing sedimentary DOM in the deepest sector of Earth's surface. *Mar Chem* 2021;**237**:104033.
5. Liu RL, Wang L, Liu Q, et al. Depth-Resolved Distribution of Particle-Attached and Free-Living Bacterial Communities in the Water Column of the New Britain Trench. *Front Microbiol* 2018;**9**:625.
6. Bolger AM, Lohse M, Usadel B. Trimmomatic: a flexible trimmer for Illumina sequence data. *Bioinformatics* 2014;**30**:2114-2120.
7. Kopylova E, Noé L, Touzet H. SortMeRNA: fast and accurate filtering of ribosomal RNAs in metatranscriptomic data. *Bioinformatics* 2012;**28**:3211-3217.
